# Predictive Value of Physiological Values and Symptom Scores for Exacerbations in Bronchiectasis and Chronic Obstructive Pulmonary Disease With Frequent Exacerbations: Longitudinal Observational Cohort Study

**DOI:** 10.2196/44397

**Published:** 2024-10-08

**Authors:** Thomas Llewelyn Jones, Claire Roberts, Scott Elliott, Sharon Glaysher, Ben Green, Janis K Shute, Anoop J Chauhan

**Affiliations:** 1 Department of Respiratory Medicine Portsmouth Hospitals University NHS Trust Portsmouth United Kingdom; 2 School of Pharmacy and Biomedical Science University of Portsmouth Portsmouth United Kingdom; 3 Translational Research Laboratory Portsmouth Hospitals University NHS Trust Portsmouth United Kingdom

**Keywords:** COPD, chronic obstructive pulmonary disease, bronchiectasis, predictive models, airway disease, symptom score

## Abstract

**Background:**

COPD (chronic obstructive pulmonary disease) and bronchiectasis are common, and exacerbations contribute to their morbidity and mortality. Predictive factors for the frequency of future exacerbations include previous exacerbation frequency and airway colonization. Earlier treatment of exacerbations is likely to reduce severity.

**Objective:**

This study tested the hypothesis that, in a population with bronchiectasis, COPD, or both who have frequent exacerbations and airway colonization, changes in symptom scores or physiological variables within 10 days prior to an exacerbation would allow the prediction of the event.

**Methods:**

We performed a 6-month, longitudinal, observational, cohort study among 30 participants with bronchiectasis, COPD, or both; at least 2 exacerbations per year; and colonization with *Pseudomonas aeruginosa* or *Haemophilus influenzae*. Daily symptom and physiological data were collected, comprising pulse rate, blood pressure, oxygen saturation, peak flow rate, step count, weight, and temperature. Exacerbations (defined as the onset of new antibiotic use for respiratory symptoms) were collected, and predictive values for abnormal values in the 10 days prior to an exacerbation were calculated.

**Results:**

A total of 30 participants were recruited, collecting a total of 39,534 physiological and 25,334 symptom data points across 5358 participant-days; these included 78 exacerbations across 27 participants, with the remaining 3 participants not having exacerbations within the 6-month observation period. Peak flow rate, oxygen saturation, and weight were significantly different at the point of exacerbation (all *P*<.001), but no significant trends around exacerbation were noted and no clinically beneficial predictive value was found in the overall or individually adjusted model. Symptom scores tended to worsen for 10 days on either side of an exacerbation but were of insufficient magnitude for prediction, with area under the receiver operating characteristic curve values of ranging from 0.4 to 0.6.

**Conclusions:**

Within this small cohort with bronchiectasis, COPD, or both and airway colonization, physiological and symptom variables did not show sufficient predictive value for exacerbations to be of clinical utility. The self-management education provided as standard of care may be superior to either of these approaches, but benefit in another or larger cohort cannot be excluded.

**International Registered Report Identifier (IRRID):**

RR2-10.2196/resprot.6636

## Introduction

COPD (chronic obstructive pulmonary disease) and bronchiectasis are common causes of morbidity and mortality across the world [1,2]. Much of this morbidity and mortality is associated with exacerbations—acute deteriorations of the disease [3,4]. Exacerbations often require additional treatment, up to and including hospitalization, and are a risk factor for progressive disease [5,6]. Certain factors are known to be associated with increased risk of future exacerbations, including recurrent previous exacerbation [7,8] and airway colonization with organisms such as *Pseudomonas aeruginosa* [9,10].

It is thought that earlier treatment of exacerbations is associated with improved outcomes, including time to recovery and reduced risk of a severe exacerbation [11], but overtreatment risks side effects from the treating medications (eg, corticosteroids or antibiotics). Accurate prediction of exacerbations is therefore required. Predictive factors may be physiological (eg, heart rate or oxygen saturations) or symptomatic (eg, degree of breathlessness or fatigue). The monitoring of physiological variables has shown value in predicting mortality in hospital and prehospital care (eg, National Early Warning Score [12,13]), including mortality in patients with COPD [14]. However, the same has not been consistently demonstrated for other metrics, such as exacerbations of disease, and previous studies focusing on lung function monitoring have not demonstrated predictive benefit [15,16]. Multiple predictors of exacerbation risk exist [17-20], such as the COPD Assessment Test score for COPD and the FACED score for bronchiectasis, but these do not help predict individual exacerbations. In order to predict exacerbations in either physiological or symptom modalities, monitoring must be remote and performed in the home environment.

We conducted a longitudinal, observational, cohort study examining the capability of physiological and symptom variables to predict exacerbations of airway disease in participants with chronic bronchitis with frequent exacerbation and airway colonization with *P aeruginosa* or *Haemophilus influenzae.* We hypothesized that changes in symptom scores or physiological variables within 10 days prior to an exacerbation would allow the prediction of the event.

## Methods

### Study Design and Setting

We conducted a 6-month, blinded, longitudinal, observational, cohort study, recruiting 30 adult participants from secondary care with a diagnosis of COPD, bronchiectasis, or both. Participants were recruited from a secondary care clinic during 2014, and the study ran from September 2014 for 6 months.

### Participants

Participants were required to have had a least 2 exacerbations in the last 12 months, at least 1 of which was within the last 6 months. Participants were required to be colonized by *P aeruginosa* or *H influenzae*, demonstrated by at least 2 cultures at exacerbation in the last 12 months without culture of the other organism. Participants were required to be able to give informed consent, comply with study procedures, and produce at least 5 mL of sputum most days. A full study protocol has been previously published [21], and the participant flow diagram is shown in the Figure S1 in Multimedia Appendix 1.

### Variables

At enrollment, participants provided clinical history, underwent spirometry testing, and completed symptom questionnaires including the St. George’s Respiratory Questionnaire (SGRQ). A home visit was conducted when participants were provided with home physiological monitoring equipment [21], specifically a digital peak flow meter, pulse oximeter, physical activity (step) tracker, infrared thermometer, automatic sphygmomanometer, and weighing scales (see Table S1 in Multimedia Appendix 1 for the models of the equipment); they were linked by Bluetooth to an iPad, which was also used collected symptom scores on a 10-point visual analog scale for appetite, breathing, cough, energy, and wellness. These data were transmitted daily to a secure cloud-based database, and the study team were blinded to the data until all participants had completed the study, except for a single unblinded technical observer who ensured that data were being received from each participant. Participants were asked to record whether they had started a course of antibiotics at home (defined as a moderate exacerbation as per guidelines) or whether they were in hospital (defined as a severe exacerbation) [22].

### Outcomes

The outcome under investigation was the accuracy of abnormal values of the physiological and symptom variables listed above to predict a moderate exacerbation of airway disease, as defined by patient-reported initiation of antibiotics for a worsening in respiratory symptomatology. Independent variables were therefore symptom scores (appetite, breathing, cough, energy, and wellness) and physiological values (heart rate, blood pressure, peripheral oxygen saturation, temperature, weight, and daily step count).

### Study Size

To establish an association between predictive markers and the occurrence of exacerbations, the study was designed around the identification of a presymptomatic period. Based on clinical expertise and literature review, we determined that a 10-day window before the onset of an exacerbation is a critical timeframe in which deviations in predictive markers can be most reliably attributed to a forthcoming exacerbation. This period selection aligns with the natural history of exacerbations as detailed in prior studies. Given the adoption of a 10-day cycle as a unit of observation, we anticipated a minimum of 12 such units per participant across an expected follow-up duration of 4 months. This frequency follows from the inclusion criterion requiring participants to have had at least 2 exacerbations in the previous year, paralleling the exacerbation frequency described by Seemungal et al [23].

For the study to hold clinical relevance, we aimed to achieve a positive predictive value (PPV) of 60% and a negative predictive value (NPV) of 90%. This was based on the study team’s experience rather than published data. On the assumption that each participant would experience at least 1 exacerbation during the study period and using a significance threshold of 5% with 90% statistical power to discern the prescribed PPV and NPV, the initial calculation suggested the need for 120 time units of observation.

However, to address the statistical challenge posed by the nonindependence of repeated measurements from individual participants—each contributing multiple observation periods—an intraclass correlation coefficient of 0.18 was used to adjust for within-participant correlation. The resulting design effect, reflecting this lack of independence, was calculated to be approximately 3. This design effect was used to multiply the basic sample size estimate, leading to a final requirement of 360 observational time periods. Consequently, a total cohort of 30 participants would satisfy this criterion, meeting the robust statistical power necessary for the intended analyses.

### Statistical Methods

Data analysis was conducted using SPSS (version 28; IBM Corp), alongside visualization in Tableau Desktop (Tableau Software) and data management in Excel 365 (Microsoft). Descriptive statistics were used to characterize the study data, using mean and SD for normally distributed variables and median and IQR for nonnormally distributed variables. The determination of normality was guided by kurtosis and skewness indices, with thresholds set at absolute values less than 1 for normal distribution.

Comparative analyses between groups were executed using the Student *t* test (2-tailed) for normally distributed data and the Mann-Whitney *U* test for data that deviated from normal distribution. The onset of an exacerbation was defined as the day on which a participant initiated an antibiotic treatment course; any subsequent antibiotic courses beginning within 10 days of the initial course’s end were considered part of the ongoing exacerbation and not as discrete events.

The monitoring tool, a modified National Early Warning Score, was calculated with the exclusion of respiratory rate, as it could not be accurately measured remotely with the equipment provided to participants. Data were assessed longitudinally to establish individualized normal ranges for each participant by calculating variable SDs from the participant’s average, while omitting any data from a 20-day span encompassing each exacerbation (10 days before and 10 days after).

An abnormal value occurring within the 10-day presymptomatic window preceding an exacerbation was classified as a true positive. Conversely, abnormal values within 10 days following an exacerbation were designated as “late” positives. Abnormal values outside of these windows were flagged as false positives. For the negative results, days featuring no abnormal values were declared true negatives unless they fell within a 10-day period preceding an exacerbation; in such cases, they were categorized as false negatives. We defined “episode sensitivity” for each variable as the detection of at least 1 abnormal result outside of the participant's normal range within the pre-exacerbation period.

### Ethical Considerations

Ethical approval was given by the NHS South Central Research Ethics Committee (14/SC/0298), and all participants gave written, informed consent. Participants were allowed to keep study equipment at the completion of the study but there was no financial compensation. Data were anonymized for analysis to safeguard participant information. Anonymized data can be made available for suitable studies on written request.

## Results

### Overview

A total of 30 participants were recruited, with study population baseline data shown in Table 1. A CONSORT (Consolidated Standards of Reporting Trials) diagram of study recruitment is shown in Figure S1 in Multimedia Appendix 1. A total of 39,534 physiological and 25,334 symptom data points were collected across 5358 participant-days. A total of 78 exacerbations were reported during the 6-month study period by 27 (90%) of 30 participants, with the median exacerbation count being 3 (IQR 1-3.75), and the remaining 3 (10%) participants not having exacerbations during the study period. During the study, 4 participants recorded a total of 6 hospital admissions for respiratory symptoms, giving an overall annualized rate of 0.2 per year compared to 0.6 per year (17 emergency hospital attendances from 7 participants) in the year prior to admission. Participant-level physiological and symptom data showed high interindividual differences around the point of exacerbation, as shown in Figures S2 and S3 in Multimedia Appendix 1.

**Figure 1:**
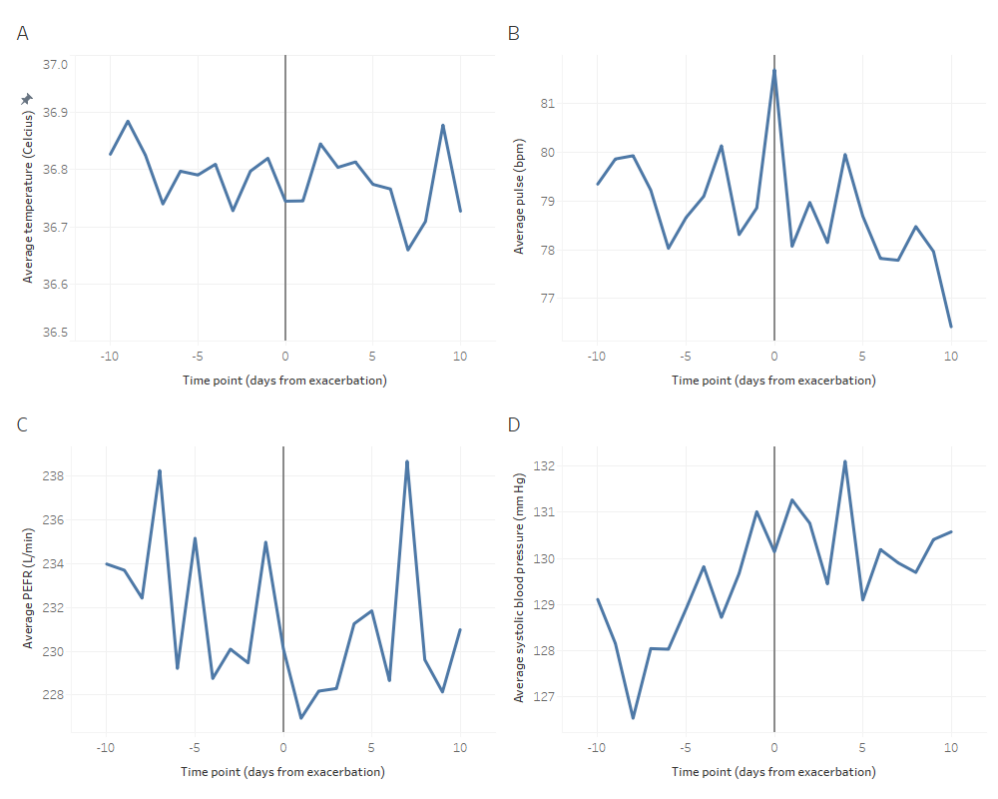
Average (A) temperature, (B) pulse rate, (C) peak expiratory flow rate (PEFR), and (D) systolic blood pressure over 10 days on either side of the start of an exacerbation for all 78 exacerbations. bpm: beats per minute.

Physiological data (Table 2) were compared using individualized *z* scores to adjust for interparticipant differences in exacerbation frequency. Values from exacerbation and 10 days on either side were compared with nonexacerbation days outside of this window. Peak flow rates and oxygen saturations were significantly lower during the exacerbation window, whereas weight and modified National Early Warning Score were significantly higher (all *P*<.001). Mean values of temperature, pulse rate, peak expiratory flow rate, and systolic blood pressure around exacerbation are shown in Figure 1.

Average appetite, breathing, cough, energy, and wellness symptom scores in the first week of data collection were correlated with overall SGRQ, giving correlation coefficients of –0.694, –0.761, –0.718, –0.798, and –0.805, respectively (all *P*<.001), showing good correlation between patient-reported symptom scores and a validated quality-of-life questionnaire. Symptom scores were also compared using individualized *z* scores to adjust for interparticipant differences in exacerbation frequency. Values from exacerbation and 10 days on either side were compared with nonexacerbation days outside of this window. All symptom scores including the total symptom score were significantly lower at and around exacerbation compared to outside this range, as shown in Table 3 (*P*<.001 for each).

**Figure 2:**
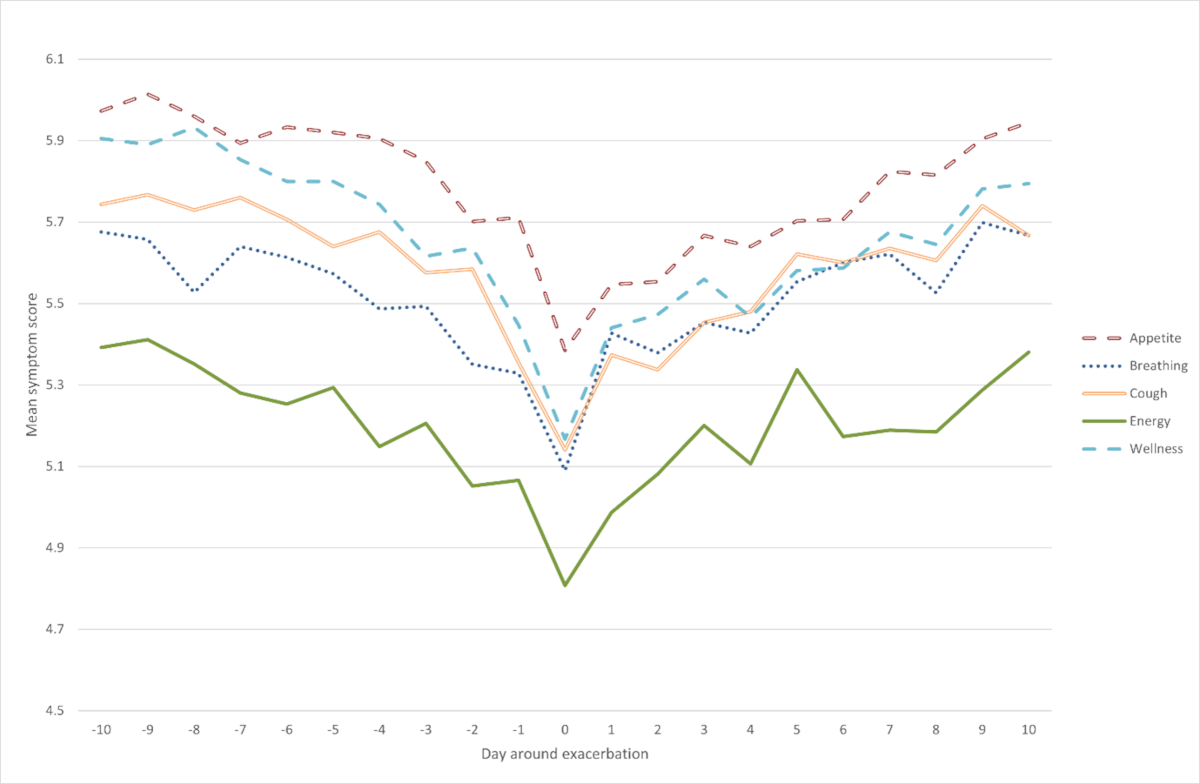
Mean symptom scores around the point of exacerbation (day 0) for all exacerbations.

The trend of symptom scores around the point of exacerbation are shown in Figure 2, showing a gradual worsening in mean symptom score each day leading to the point of exacerbation, with gradual recovery following this. However, the magnitude of the decline is small, with each score decreasing by a mean of less than 1 point and by a similar magnitude.

**Figure 3:**
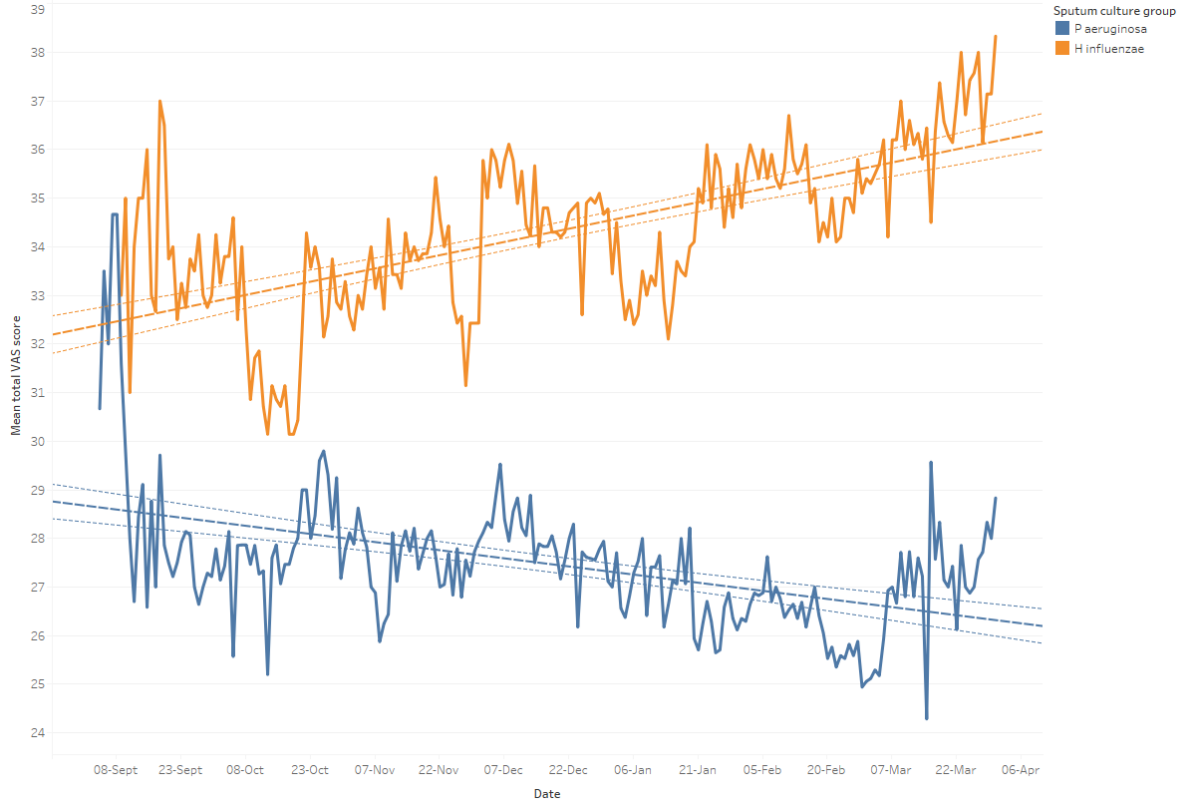
Change in mean total symptom score by sputum culture, demonstrating worsening symptoms over time in the Pseudomonas aeruginosa group (lower line, n=20) and improving symptoms in the Haemophilus influenzae group (upper line, n=10). VAS: visual analog scale.

The trends of total symptom scores through the study were examined. While the average total symptom score showed a slight improvement over time, this was not true for those with sputum *P aeruginosa*, who started with poorer symptom scores and showed a trend toward worsening over time, as shown in Figure 3.

**Table 1:** Study population baseline data. All participants with chronic obstructive lung disease (COPD) had a GOLD (Global Initiative for COPD) grade of D.

Variable		Value (N=30)
Participants, n (%)		30 (100)
Age (years), median (IQR)		68.3 (61.3-73.6)
Female gender, n (%)		17 (57)
**Smoking status, n (%)**		
	Never smoked	12 (40)
	Ex-smoker	17 (57)
	Current smoker	1 (3)
Pack-year history, median (IQR)		15.5 (0-30)
Antibiotic courses per participant in the past 12 months, median (IQR)		4 (3-5)
BMI (kg/m^2^), mean (SD)		26.3 (5.6)
Bronchiectasis, n (%)		17 (57)
COPD, n (%)		4 (13)
Bronchiectasis and COPD, n (%)		9 (30)
Ischemic heart disease, n (%)		4 (13)
Heart failure, n (%)		4 (13)
Type 2 diabetes, n (%)		2 (7)
FEV1^a^ (% predicted), mean (SD)		66.1 (28.4)
FVC^b^ (% predicted), mean (SD)		85 (23.4)
FEV1/FVC ratio, mean (SD)		62.6 (16.0)
Sputum culture *Pseudomonas*, n (%)		20 (67)

^a^FEV1: forced expiratory volume in 1 second.

^b^FVC: forced vital capacity.

**Table 2:** Average results for physiological data throughout the study. *P* values were calculated from 2-tailed *t* test of the exacerbation period versus the nonexacerbation period for normally distributed data or Mann-Whitney U test for nonnormally distributed data. Exacerbation data were based on 27 participants, as 3 participants did not have an exacerbation.

Physiological data	All data (n=30)	Exacerbation period (–10 to +10 days; n=27)	Normal range excluding the exacerbation period (n=27)	*P* value
Weight (kg), mean (SD)	72.7 (13.9)	74.5 (14.2)	72.2 (14.2)	<.001
Step count (per day), median (IQR)	3065 (1242-7088)	3296 (1285-6428)	2975 (1183-7469)	.48
Peak flow (L/min), median (IQR)	233 (149-320)	209 (133-289)	243 (159-327)	<.001
Oxygen saturation (%), median (IQR)	95.4 (92.0-97.2)	95.2 (92.0-97.0)	95.5 (92.5-97.3)	<.001
Systolic BP^a^ (mm Hg), mean (SD)	130 (22)	130 (24)	130 (22)	.90
Diastolic BP (mm Hg), mean (SD)	77 (12)	77 (13)	77 (12)	.88
Pulse rate (per min), mean (SD)	78.4 (13.2)	78.8 (14.1)	78.2 (11.9)	.052
Temperature (^o^C), median (IQR)	36.7 (36.5-37.0)	36.7 (36.5-37.0)	36.6 (36.5-37.0)	.008
mNEWS^b^, mean (SD)	1.4 (1.6)	1.5 (1.7)	1.3 (1.6)	<.001

^a^BP: blood pressure.

^b^mNEWS: modified National Early Warning Score.

**Table 3:** Average results for all symptom data throughout the study and excluding exacerbations. *P* values were calculated from 2-tailed *t* test of the exacerbation period versus the nonexacerbation period.

Symptom scores	All data (n=30)	Exacerbation period (–10 to 10 days; n=27)	Normal range excluding the exacerbation period (n=27)	*P* value
Wellness score, mean (SD)	6.1 (1.8)	5.7 (18)	6.4 (1.8)	<.001
Cough score, mean (SD)	6.1 (1.8)	5.6 (1.7)	6.4 (1.8)	<.001
Breathing score, mean (SD)	6.0 (2.0)	5.5 (2.0)	6.2 (1.9)	<.001
Appetite score, mean (SD)	6.2 (2.0)	5.8 (2.0)	6.4 (2.0)	<.001
Energy score, mean (SD)	5.8 (2.1)	5.2 (2.0)	6.0 (2.1)	<.001
Total symptom score, mean (SD)	30.1 (9.2)	27.7 (8.9)	31.4 (9.1)	<.001

### Predictive Values

Individual control limits of variable widths were calculated as above. Table 4 shows predictive values at 2 SDs, which gave a balance between PPV and sensitivity. A range of control interval widths were calculated, and PPV for physiological variables were highest at 27.9% (at 3.3 SDs), while PPV for symptom variables peaked at 62.4% (at 4 SDs), which predicted 17.6% and 26.1% of episodes, respectively (Figures S4 and S5 in Multimedia Appendix 1). Individual-level data demonstrating the range of predictive values at control widths of 2 SDs for each participant are shown in Table S2 in Multimedia Appendix 1. Comparison between results for participants with COPD and bronchiectasis is shown in Table S3 in Multimedia Appendix 1, showing slightly higher PPV and episode sensitivity for participants with COPD but still less than 50%.

Physiological and symptom variables were analyzed using receiver operating characteristic analysis (Figure S6 in Multimedia Appendix 1), and no area under the curve (AUC) values were outside the range of 0.4-0.6, indicating no significant predictive value. This was repeated with *z* scores (Figures S7 and S8 in Multimedia Appendix 1) to normalize for individual variation, and all AUC values were within the 0.45-0.55 range, again showing no significant predictive value (Figure S9 in Multimedia Appendix 1). Receiver operating characteristic analysis was also conducted for COPD and bronchiectasis alone and for *P aeruginosa* and *H influenzae* colonization alone; in each analysis, no curve exceeded an AUC of 0.4-0.6, with the exception of peak flow in participants with *H influenzae,* which had an AUC of 0.374.

**Table 4:** Predictive values of abnormal results for physiological and symptom results with individualized control limits of 2 SDs.

Physiological and symptom variables	PPV^a^ (%)	NPV^b^ (%)	Sensitivity (%)	Specificity (%)	Accuracy (%)	Episode sensitivity (%)
Weight	27.23	82.76	7.26	95.82	80.14	24.36
Steps	13.51	82.51	3.46	95.36	79.45	19.23
Peak flow rate	26.18	83.06	6.90	95.91	80.46	28.21
Oxygen saturation	24.63	83.17	6.80	95.68	80.39	33.33
Systolic BP^c^	21.39	82.74	5.82	95.48	79.83	35.90
Pulse rate	20.00	82.94	5.44	95.48	79.99	37.18
Temperature	24.41	82.99	6.99	95.45	80.07	42.31
Wellness score	36.88	83.98	12.99	95.35	81.11	34.62
Cough score	36.81	84.28	16.06	94.22	80.69	42.31
Breathing score	37.19	84.51	18.07	93.61	80.52	43.59
Appetite score	40.29	84.27	14.99	95.35	81.44	47.44
Energy score	38.44	84.44	17.14	94.25	80.89	46.15
Total symptom score	38.46	84.28	15.39	94.85	81.11	43.59

^a^PPV: positive predictive value.

^b^NPV: negative predictive value.

^c^BP: blood pressure.

## Discussion

Our study has revealed the limited predictive value of physiological markers and symptom scores in this population with airway disease and chronic colonization. The variability of physiological data and the subtlety of symptom changes challenge the reliance on these measures alone for predicting imminent exacerbations. Symptom scores on a population level indicated some deterioration around the time of exacerbation but were insufficient as stand-alone individual level predictors. Our approach to personalize reference ranges, accounting for intraparticipant variability, has been proven valuable but still fell short in enhancing predictive accuracy.

Despite these limitations, the role of these indicators cannot be entirely discounted. Physiological variables and symptom scores remain crucial components of a comprehensive disease monitoring plan for many respiratory diseases. The differentiation between physiological changes and symptom deterioration is important; while physiological parameters may not predict exacerbations with high accuracy, they provide valuable information on a patient’s baseline health status, which can be crucial when responding to symptoms that suggest an exacerbation and therefore guide clinical decision-making, particularly when considered alongside an individual’s typical symptomatology and exacerbation patterns. This approach was used in the PROMETE study for example, which showed that monitoring physiological values with respiratory physician reviews reduced hospital and emergency department attendances over 7 months, with more exacerbations being managed at home [24].

Moreover, the aggregated symptom score highlighted in our study, while not overtly predictive, could have potential applications when considering temporal trends over more extended periods, as some data suggest that telemonitoring is more beneficial over a longer term than in our study [25]. While acute predictive value is limited, monitoring this score could be useful for assessing overall disease management and quality of life across broader timeframes, which may be of interest in longitudinal studies. Systematic reviews of previous studies of the effectiveness of remote monitoring of airway disease have focused on COPD and have shown mixed results [26,27]. Of particular interest, physiological deterioration over time was noted in our study in those colonized by *P aeruginosa*, and this is consistent with previously reported data, suggesting a particularly high-risk group of patients [28].

Our study also reflects the multifaceted nature of exacerbations in chronic airway diseases, which likely result from a combination of factors, ranging from environmental triggers to individual patient behaviors. Furthermore, the findings underscore the essential role of education and self-management. The decrease in admission rates during the study, compared to the year prior, may indicate that equipping patients with self-management plans may help avert the most severe manifestations of exacerbations, thereby reducing hospital visits.

While our study has provided valuable insights into the management of chronic airway disease, the inherent limitations must be acknowledged. The relatively small cohort size and the specific inclusion of patients with known chronic airway colonization restrict the generalizability of the findings. The observational period did not extend across a full year, resulting in a potential underrepresentation of seasonal variations, although it crucially encompassed the winter months when exacerbations are generally more prevalent and severe. The exclusion of respiratory rate—a potentially significant physiological marker—from our data collection may have omitted a critical variable with predictive capability. Additionally, while the symptom scores used in the study were less complex and showed good correlation with established measures such as the SGRQ, there remains the possibility that more nuanced symptom evaluation tools could yield stronger predictive correlations. Another notable aspect of the study was participant access to their own longitudinal data, which introduces the potential for reporting bias. However, the anticipated direction of such bias would more commonly lead to an overestimation of symptoms, resulting in false-positive identifications of exacerbations—a phenomenon not observed in our data. This suggests that while participant awareness of their data could be a confounding factor, its impact on the study’s outcomes may be minimal. Despite these limitations, the study’s strengths also warrant mention. By methodically tracking daily symptomatic and physiological changes within a clearly defined patient group, the study provides nuanced insight into the patterns preceding exacerbations. Moreover, the inclusion of the winter months offers pertinent data from a period of high clinical relevance due to the increased exacerbation risk.

Further studies examining respiratory rate, other symptom assessment, or continuous monitoring may be of benefit, but a strongly predictive single factor seems unlikely and alternative approaches to monitoring airway disease such as combining multiple sources of predictive data in the home setting are more likely to be useful.

## Appendix

Multimedia Appendix 1 Supplementary tables and figures.

## Abbreviations

AUC: area under the curve
CONSORT: Consolidated Standards of Reporting Trials
COPD: chronic obstructive pulmonary disease
NPV: negative predictive value
PPV: positive predictive value
SGRQ: St. George’s Respiratory Questionnaire
