# Supplementary material for: Predictive Value of Physiological Values and Symptom Scores for Exacerbations in Bronchiectasis and Chronic Obstructive Pulmonary Disease With Frequent Exacerbations: Longitudinal Observational Cohort Study
Source: Interact J Med Res. 2024 Oct 8;13:e44397. doi: 10.2196/44397 (PMC11496917; doi:10.2196/44397)
Supplement: Multimedia Appendix 1 [file ijmr_v13i1e44397_app1.docx]

**Multimedia Appendix 1.** Supplementary data and figures.


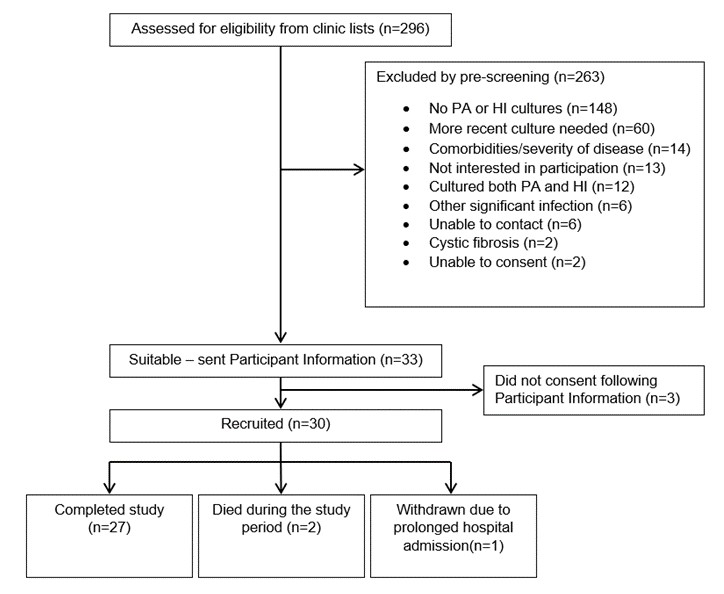


**Figure S1.** CONSORT diagram of study recruitment


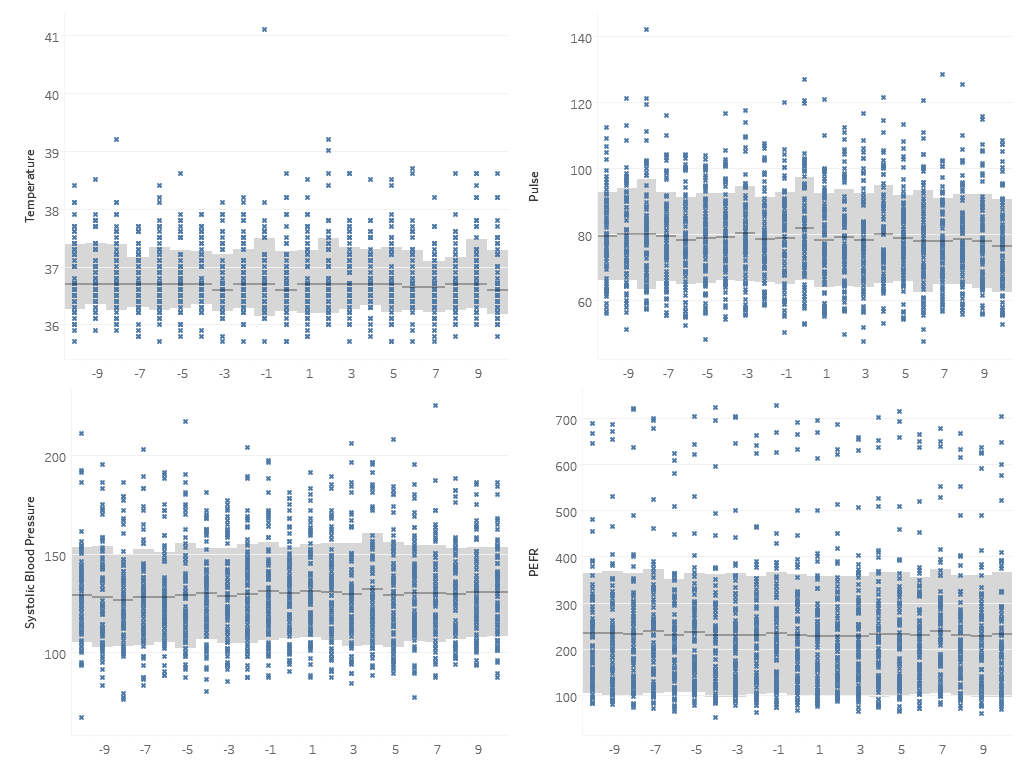


**Figure S2.** Individual data points for physiological variables from -10 to +10 days around exacerbation showing mean (line) and ±1SD (grey area) demonstrating the lack of consistent change to the point of exacerbation and the high variation around the mean


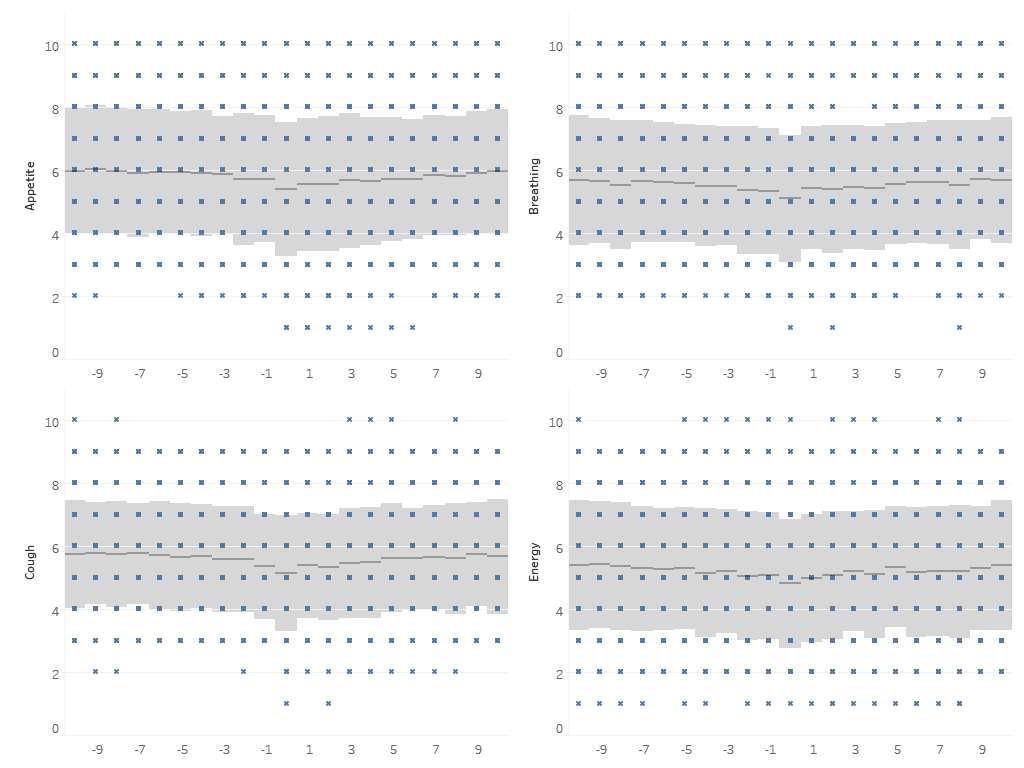


**Figure S3.** Individual data points for symptom data from -10 to +10 days around exacerbation showing mean (line) and ±1SD (grey area), demonstrating the slight average reduction to the point of exacerbation but the high variation around the mean


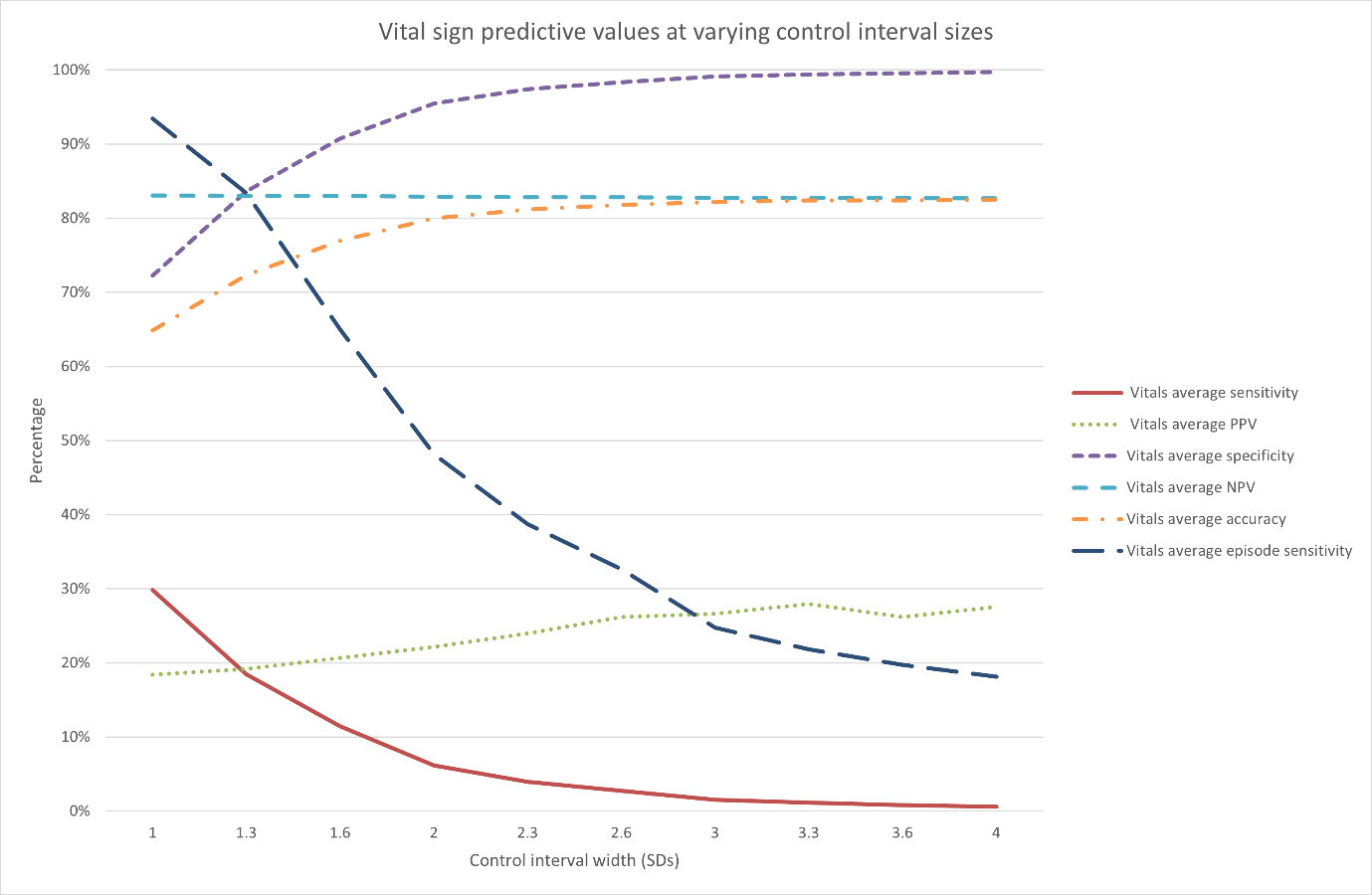


**Figure S4.** Mean predictive values for physiological data (weight, daily step count, peak flow rate, oxygen saturation, heart rate, systolic blood pressure, temperature, and total modified NEWS) as the control interval width is varied. This demonstrates an increase in specificity as the control intervals widen but a subsequent drop in sensitivity and generally poor positive predictive values.


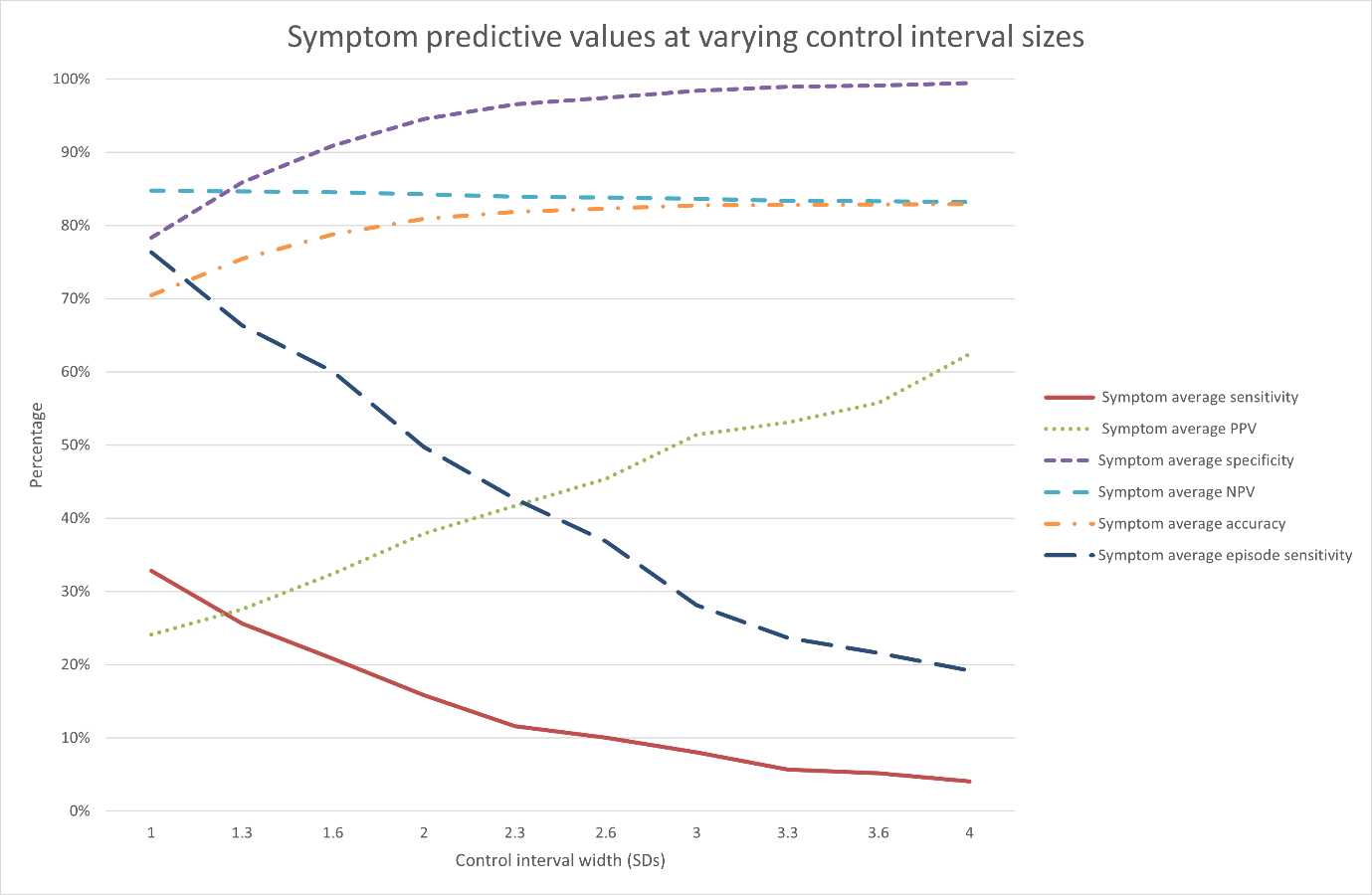


**Figure S5.** Mean predictive values for symptom data (10-point likert-type scales for appetite, breathing, cough, energy and wellness) as the control interval width is varied. This demonstrates a peak positive predictive value of over 60%, but very low sensitivity at this point.


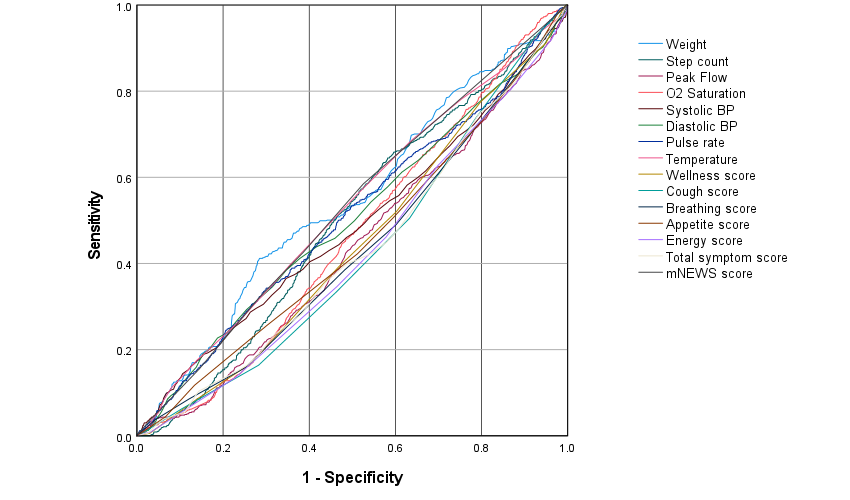


**Figure S6.** ROC curve for unadjusted physiological and symptom data to predict exacerbation within 10 days prior to onset


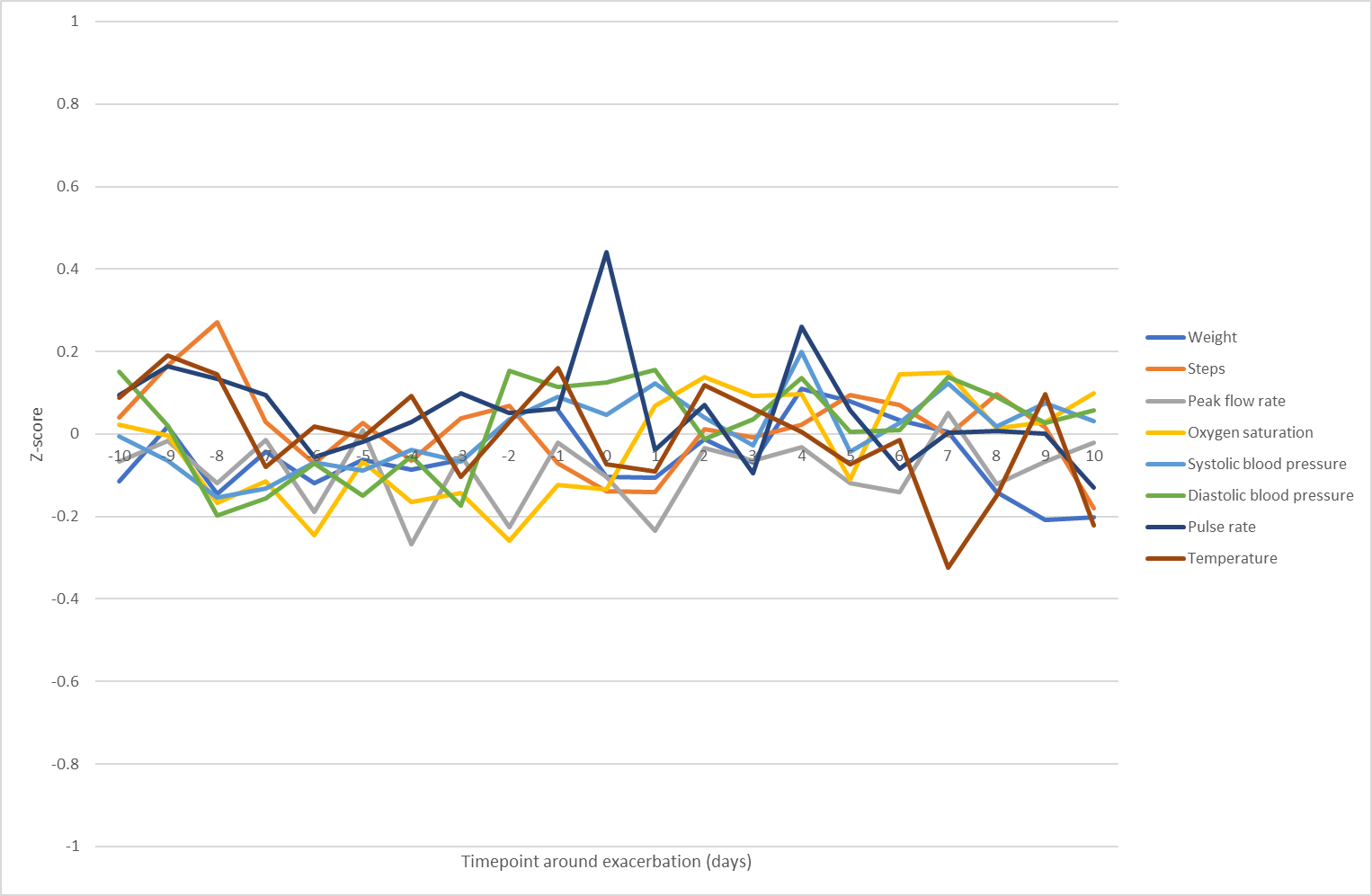


**Figure S7.** plot of z-scores of physiological data from 10 days prior to 10 days after an exacerbation


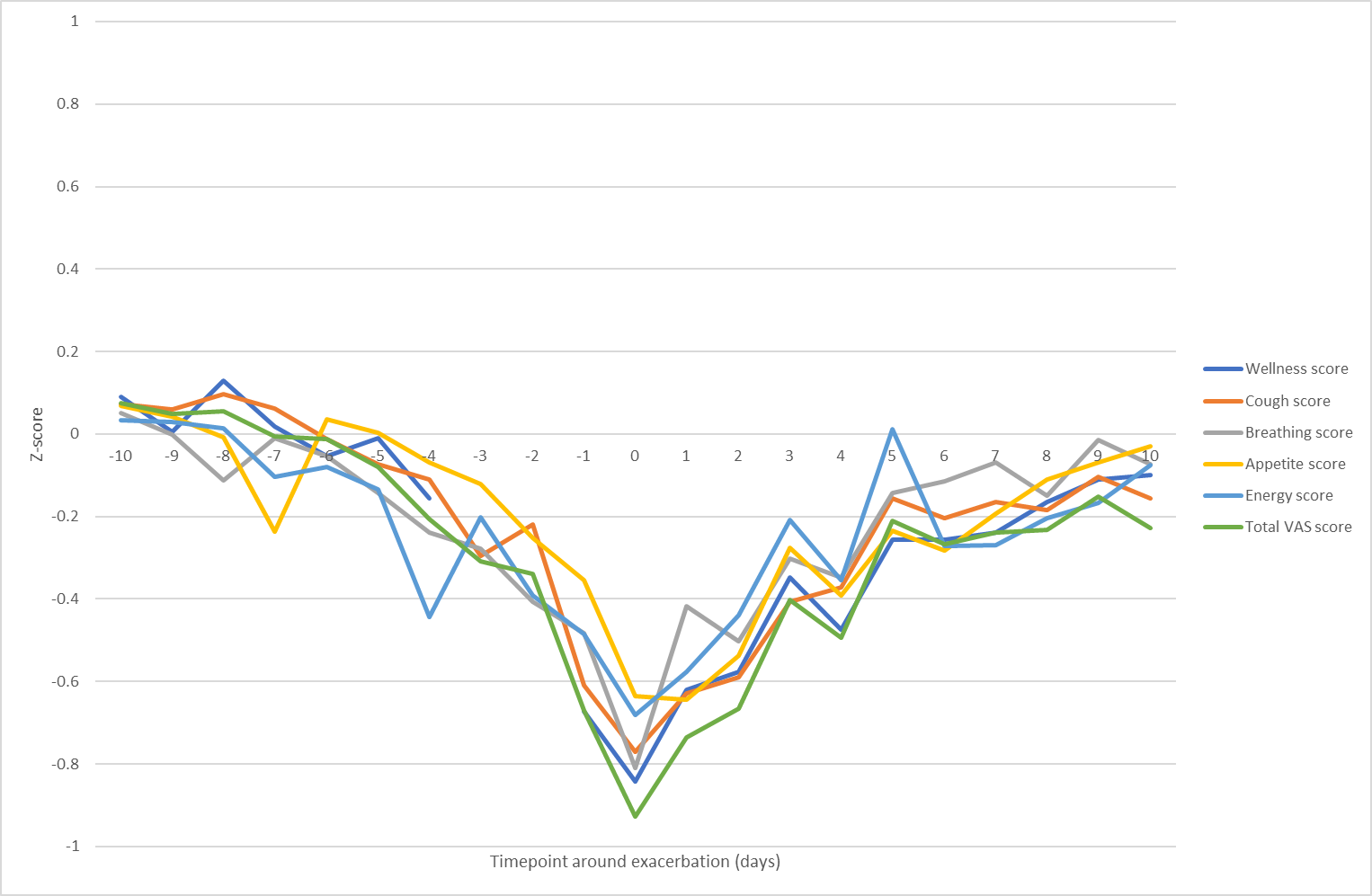


**Figure S8.** plot of z-scores of VAS symptom scores from 10 days prior to 10 days after an exacerbation


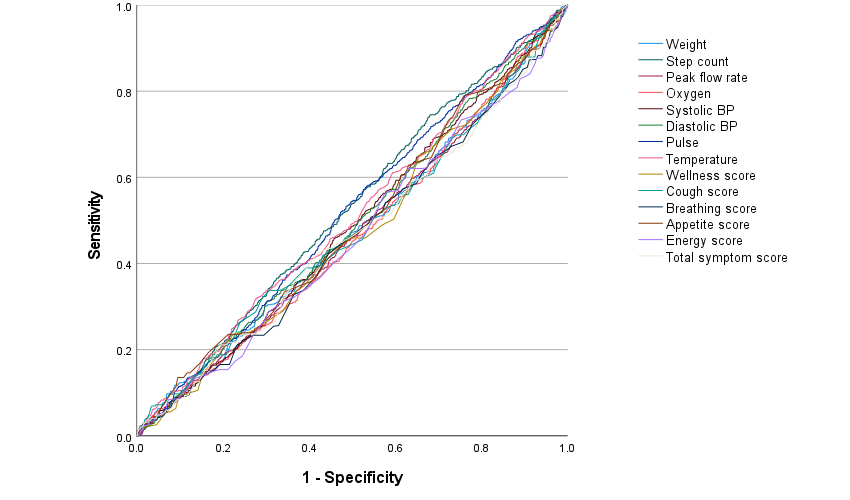


**Figure S9.** ROC curve for z-scores of physiological and symptom data to predict exacerbation within 10 days prior to onset.

| **Equipment** | **Make** |
| --- | --- |
| Pulse oximeter | Pulse Oximeter, Activ8rlives, UK |
| Digital peak flow meter | Clement Clarke Mini-Wright Digital Peak Flow Meter, Clement Clarke International Ltd, UK |
| Physical activity (step) tracker | BuddyBand, Activ8rlives, UK |
| Infrared thermometer | Contactless Thermometer, Activ8rlives, UK |
| Automatic sphygmomanometer | Activ8rlives Blood Pressure Monitor, Activ8rlives, UK |
| Weighing scales | Body Analyser Smart Scales, Activ8rlives, UK |
| iPad | iPad mini, Apple Inc., USA |

**Table S1.** Participant study equipment.

|  | **PPV** | **NPV** | **Sensitivity** | **Specificity** | **Accuracy** | **Episode Sensitivity** |
| --- | --- | --- | --- | --- | --- | --- |
| Weight | 13.8% (00.0%-45.0%) | 82.3% (76.5%-92.9%) | 03.3% (00.0%-11.1%) | 97.0% (94.3%-98.0%) | 81.2% (74.1%-89.0%) | 25.0% (00.0%-40.0%) |
| Steps | 00.0% (00.0%-20.0%) | 81.6% (72.1%-91.2%) | 00.0% (00.0%-10.0%) | 95.6% (93.9%-97.3%) | 77.4% (71.3%-86.1%) | 00.0% (00.0%-50.0%) |
| PEFR | 25.0% (00.0%-38.9%) | 83.9% (76.9%-88.0%) | 03.3% (00.0%-10.0%) | 96.0% (95.0%-97.1%) | 81.2% (75.2%-86.2%) | 25.0% (00.0%-50.0%) |
| Oxygen Sats | 16.7% (00.0%-40.0%) | 83.3% (75.8%-90.5%) | 03.3% (00.0%-07.7%) | 95.6% (94.4%-97.3%) | 81.5% (75.0%-87.4%) | 33.3% (00.0%-50.0%) |
| Systolic BP | 14.3% (00.0%-37.5%) | 81.5% (74.1%-90.6%) | 05.0% (00.0%-10.0%) | 95.7% (94.3%-96.5%) | 79.9% (71.7%-86.5%) | 33.3% (00.0%-66.7%) |
| Pulse rate | 20.0% (00.0%-33.3%) | 82.3% (76.1%-90.8%) | 04.8% (00.0%-07.4%) | 95.5% (94.9%-96.7%) | 78.5% (74.2%-88.4%) | 33.3% (00.0%-50.0%) |
| Temperature | 16.7% (00.0%-33.3%) | 81.3% (77.3%-91.6%) | 10.0% (03.3%-10.0%) | 95.5% (94.4%-97.0%) | 79.5% (72.7%-88.2%) | 50.0% (33.3%-75.0%) |
| mNEWS | 14.3% (00.0%-40.0%) | 83.0% (76.5%-90.7%) | 07.1% (00.0%-11.4%) | 95.6% (94.0%-96.9%) | 81.0% (72.6%-85.9%) | 40.0% (00.0%-50.0%) |
| Wellness | 20.0% (00.0%-54.9%) | 82.8% (79.1%-90.8%) | 04.3% (00.0%-20.0%) | 96.8% (93.8%-98.6%) | 81.8% (73.9%-88.4%) | 25.0% (00.0%-50.0%) |
| Cough | 33.3% (00.0%-41.7%) | 84.7% (76.4%-92.8%) | 10.0% (00.0%-17.5%) | 96.1% (92.7%-97.7%) | 78.3% (73.9%-89.6%) | 33.3% (00.0%-66.7%) |
| Breathing | 22.0% (00.0%-43.3%) | 83.8% (79.7%-92.4%) | 06.9% (00.0%-23.3%) | 94.5% (90.2%-97.1%) | 80.7% (72.4%-87.7%) | 33.3% (00.0%-66.7%) |
| Appetite | 50.0% (11.8%-72.2%) | 84.1% (79.2%-90.9%) | 10.0% (03.3%-20.0%) | 97.0% (93.3%-98.4%) | 81.1% (77.0%-86.4%) | 50.0% (25.0%-66.7%) |
| Energy | 25.0% (00.0%-50.0%) | 85.0% (78.8%-92.9%) | 10.0% (00.0%-16.7%) | 95.5% (92.5%-97.5%) | 81.2% (75.6%-87.4%) | 40.0% (00.0%-66.7%) |
| Total VAS score | 26.8% (00.0%-53.6%) | 84.7% (78.5%-91.4%) | 06.7% (00.0%-24.1%) | 95.3% (93.3%-97.0%) | 82.1% (75.5%-89.3%) | 33.3% (00.0%-66.7%) |

**Table S2.** Predictive values for symptom and physiological variables at the individual participant level at control width intervals of 2 standard deviations.

|  | Symptom scores | | | | | | Physiological measures | | | | | |
| --- | --- | --- | --- | --- | --- | --- | --- | --- | --- | --- | --- | --- |
|  | Sensitivity | PPV | Specificity | NPV | Accuracy | Episode Sensitivity | Sensitivity | PPV | Specificity | NPV | Accuracy | Episode Sensitivity |
| Bronchiectasis | 13.89% | 34.37% | 94.88% | 85.01% | 81.70% | 39.12% | 6.31% | 21.44% | 95.50% | 83.98% | 80.98% | 37.06% |
| COPD | 19.84% | 49.16% | 93.77% | 79.93% | 76.97% | 45.50% | 5.37% | 26.83% | 95.64% | 77.29% | 74.97% | 32.00% |

**Table S3.** Comparison of mean predictive values for participants with COPD or bronchiectasis at 2 standard deviation control widths, demonstrating slightly higher PPV and episode sensitivity for participants COPD but still lower than 50%. Participants with both conditions contribute to both groups.
